# Heterozygote advantage at HLA class I and II loci and reduced risk of colorectal cancer

**DOI:** 10.3389/fimmu.2023.1268117

**Published:** 2023-10-24

**Authors:** Ya-Yu Tsai, Chenxu Qu, Joseph D. Bonner, Rebeca Sanz-Pamplona, Sidney S. Lindsey, Marilena Melas, Kevin J. McDonnell, Gregory E. Idos, Christopher P. Walker, Kevin K. Tsang, Diane M. Da Silva, Ferran Moratalla-Navarro, Asaf Maoz, Hedy S. Rennert, W. Martin Kast, Joel K. Greenson, Victor Moreno, Gad Rennert, Stephen B. Gruber, Stephanie L. Schmit

**Affiliations:** ^1^ Genomic Medicine Institute, Cleveland Clinic, Cleveland, OH, United States; ^2^ Norris Comprehensive Cancer Center, University of Southern California, Los Angeles, CA, United States; ^3^ Center for Precision Medicine, City of Hope National Medical Center, Duarte, CA, United States; ^4^ Catalan Institute of Oncology (ICO), Hospitalet de Llobregat, Barcelona, Spain; ^5^ ONCOBELL Program, Bellvitge Biomedical Research Institute (IDIBELL), Hospitalet de Llobregat, Barcelona, Spain; ^6^ Consortium for Biomedical Research in Epidemiology and Public Health (CIBERESP), Madrid, Spain; ^7^ Hospital Universitario Lozano Blesa, Aragon Health Research Institute (IISA), ARAID Foundation, Aragon Government, Zaragoza, Spain; ^8^ Molecular Diagnostics, New York Genome Center, New York, NY, United States; ^9^ Department of Clinical Sciences, Faculty of Medicine and Health Sciences and Universitat de Barcelona Institute of Complex Systems (UBICS), University of Barcelona, Barcelona, Spain; ^10^ Department of Medical Oncology, Dana-Farber Cancer Institute and Harvard Medical School, Boston, MA, United States; ^11^ B. Rappaport Faculty of Medicine, Technion and the Association for Promotion of Research in Precision Medicine (APRPM), Haifa, Israel; ^12^ Department of Pathology, University of Michigan, Ann Arbor, MI, United States; ^13^ Population and Cancer Prevention Program, Case Comprehensive Cancer Center, Cleveland, OH, United States

**Keywords:** heterozygote advantage, human leukocyte antigen, heterozygosity, HLA diversity, colorectal cancer

## Abstract

**Objective:**

Reduced diversity at Human Leukocyte Antigen (HLA) loci may adversely affect the host’s ability to recognize tumor neoantigens and subsequently increase disease burden. We hypothesized that increased heterozygosity at HLA loci is associated with a reduced risk of developing colorectal cancer (CRC).

**Methods:**

We imputed HLA class I and II four-digit alleles using genotype data from a population-based study of 5,406 cases and 4,635 controls from the Molecular Epidemiology of Colorectal Cancer Study (MECC). Heterozygosity at each HLA locus and the number of heterozygous genotypes at HLA class -I (*A*, *B*, and *C*) and HLA class -II loci (*DQB1*, *DRB1*, and *DPB1*) were quantified. Logistic regression analysis was used to estimate the risk of CRC associated with HLA heterozygosity. Individuals with homozygous genotypes for all loci served as the reference category, and the analyses were adjusted for sex, age, genotyping platform, and ancestry. Further, we investigated associations between HLA diversity and tumor-associated T cell repertoire features, as measured by tumor infiltrating lymphocytes (TILs; N=2,839) and immunosequencing (N=2,357).

**Results:**

Individuals with all heterozygous genotypes at all three class I genes had a reduced odds of CRC (OR: 0.74; 95% CI: 0.56-0.97, *p*= 0.031). A similar association was observed for class II loci, with an OR of 0.75 (95% CI: 0.60-0.95, *p*= 0.016). For class-I and class-II combined, individuals with all heterozygous genotypes had significantly lower odds of developing CRC (OR: 0.66, 95% CI: 0.49-0.87, *p*= 0.004) than those with 0 or one heterozygous genotype. HLA class I and/or II diversity was associated with higher T cell receptor (TCR) abundance and lower TCR clonality, but results were not statistically significant.

**Conclusion:**

Our findings support a heterozygote advantage for the HLA class-I and -II loci, indicating an important role for HLA genetic variability in the etiology of CRC.

## Introduction

Human leukocyte antigen (HLA) class I and II loci play an important role in adaptive and innate immunity. HLA class I (HLA-I) presents foreign antigens to cytotoxic T cells, and HLA class II (HLA-II) stimulates antibody production in response to specific antigens. Individuals who carry heterozygous genotypes at the HLA genes are able to display a greater variety of antigenic peptides than those with homozygous genotypes at the HLA genes, resulting in an immune response to a broader range of antigens (1). The heterozygote advantage, proposed by Doherty and Zinkernagel (2), suggests that individuals with greater diversity (i.e. more heterozygous genotypes) at HLA genes have better fitness by presenting a broader set of tumor antigens for T cells recognition. This phenomenon has been observed in infectious diseases and cancers associated with viral infections, such as progression of AIDS (3), HBV-associated hepatocellular carcinoma (4–6), inflammatory bowel diseases (7), and non-Hodgkin’s lymphoma (NHL) (8). For example, patients who carry heterozygous HLA class I alleles appear to have slower progression from HIV to AIDS, while subjects with heterozygotes at HLA class II loci have greater ability to clear hepatitis B virus (HBV) and hepatitis C virus (HCV) infections.

Colorectal cancer (CRC) is a complex disease, involving a series of genetic events, immune responses, and exogenous factors. Disease progression and responses to immunotherapies may vary by stage at diagnosis, microsatellite instability status, tumor mutation burden (TMB), and other factors in the tumors (9). HLA, especially HLA-I, is frequently lost in CRC tumors, resulting in tumor immune escape from cytotoxic T cells during the cancer development and progression (10). Although T cell-mediated immunotherapies have appeared as a promising regimen for several cancers including CRC, only a subset of patients are responsive to treatment. A recent study by Chowell et al. showed that germline diversity of HLA class I alleles is associated with better response to checkpoint blockade immunotherapy in patients with melanoma and non-small cell lung cancer (11, 12). These findings provide further evidence in support of the HLA heterozygote advantage hypothesis, where HLA-heterozygous individuals present a broader immunopeptidome for recognition by cytotoxic T cells.

**Figure 1:**
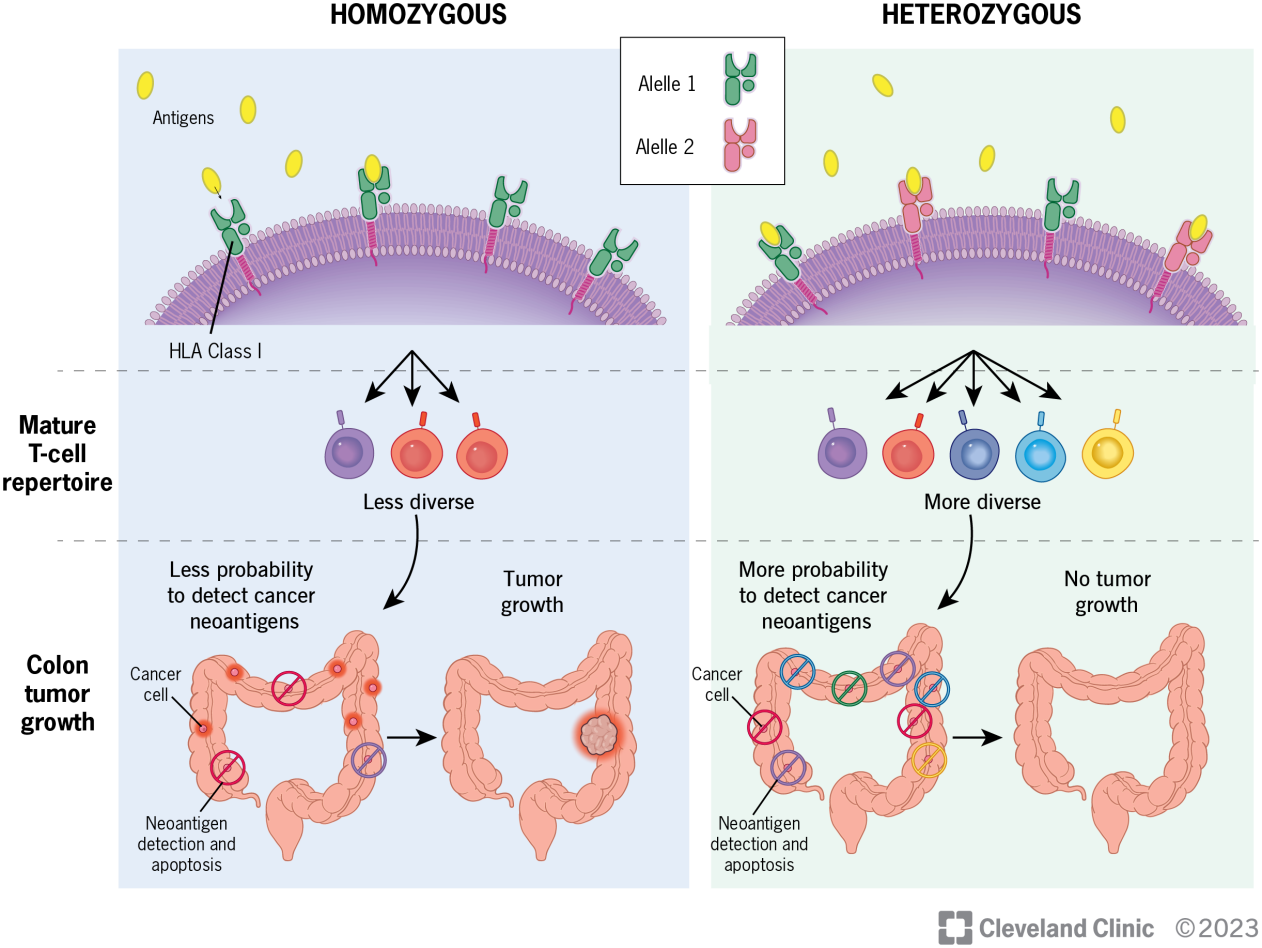
Diversity of HLA genotypes and its potential role in the development of colorectal cancer.

Given the growing evidence of the importance of HLA diversity in tumor development, progression and immunotherapy response, we proposed that increased diversity in germline HLA class I and/or II loci is associated with reduced risk of developing CRC. Heterozygous HLA genotypes may facilitate the presentation of a broader set of tumor antigens in a greater range of contexts for T cell recognition, thereby leading to early tumor elimination and reduction of CRC risk (**Figure 1**). HLA genotype, although not modifiable, may be beneficial as a biomarker to be incorporated into risk-stratified screening guidelines. To test this hypothesis, we conducted the largest population-based study to date to examine the association between diversity in HLA class I and class II loci, measured by heterozygosity, and the risk of developing CRC.

## Materials and methods

### Study population

The Molecular Epidemiology of Colorectal Cancer Study (MECC) is a population-based study of incident CRC cases and healthy controls recruited in northern Israel from 1998 through 2017. Cases include those with invasive colorectal adenocarcinoma. Controls are participants without prior history of CRC selected from the same source population as cases and with individual matching on age, gender, Jewish ethnicity, and usual clinic location. A detailed description of the study population has been described elsewhere (13). Baseline demographic and clinical characteristics of the MECC subjects contributing to this study are described in **Table 1**.

**Table 1:** Descriptive table for the Molecular Epidemiology of Colorectal Cancer (MECC) study population.

Characteristics	Cases			Controls	
N	5406			4635	
	Mean		Standard Deviation	Mean	Standard Deviation
Age	68		12.45	71	11.90
	N		**%**	N	**%**
Sex					
Male	2819		52.15	2409	51.97
Female	2587		47.85	2226	48.03
Ethnicity					
Arab	846		15.65	566	12.21
Ashkenazi	3150		58.27	2676	57.73
Other	325		6.01	223	4.81
Sephardi	1085		20.07	1170	25.24
Stage					
I	1068		19.76	–	–
II	2123		39.27	–	–
III	835		15.45	–	–
IV	535		9.90	–	–
Missing	845		15.63	–	–
MSI					
Stable	2857		52.85	–	–
Unstable	621		11.49	–	–
Missing	1928		35.66		

### Genotyping and quality control

Germline DNA samples from 10,041 MECC subjects (5,406 CRC cases and 4,635 controls) were genotyped using three genotyping platforms. 485 cases and 498 controls were genotyped in two batches using Illumina HumanOmni2.5 chips, measuring approximately 2.3 million SNPs (14). Batch 1 (384 cases and 143 controls) was genotyped at the Case Western Reserve University, and batch 2 (101 cases and 355 controls) was genotyped at the University of Michigan. 1,155 cases and 1,117 controls were genotyped using a custom Affymetrix Axiom genome-wide platform measuring 1.2 million SNPs (15). 3,768 cases and 3,028 controls were genotyped using a custom Illumina OncoArray chip measuring 495K SNPs (genome-wide backbone and known cancer susceptibility loci) (16). All genotype data were cleaned by platform using common quality control metrics at the individual level and SNP levels described previously (14–16). After QC, a total of 10,041 subjects including 5,406 cases and 4,635 controls were included.

Principal component analysis (PCA) was performed using PLINK 1.9 on directly genotyped SNPs shared across the four genotyping panels: Illumina Omni2.5, Affymetrix Axiom, Illumina Custom OncoArray, and Illumina Infinium OncoArray-500K. After LD pruning (r^2^>0.2), removing SNPs with minor allele frequency (MAF) < 0.01, and SNPs with PC1 and PC2 loading >4.0, 55,852 autosomal SNPs were retained for PCA. Due to possible residual population substructure, the first 5 principal components for global ancestry were included in association analyses.

### HLA genotype imputation

Of the directly genotyped 55,852 SNPs, we performed imputation for the HLA region using 7,727 SNPs on chromosome 6. HLA class I (*HLA-A*, *HLA-B*, and *HLA-C*) and class II (*HLA-DQA1*, *HLA-DQB1*, *HLA-DRB1*, *HLA-DPA1*, and *HLA-DPB1*) loci were imputed using SNP2HLA and a reference panel of 5,225 unrelated individuals from the Type 1 Diabetes Genetics Consortium (17). In summary, 278 classical HLA alleles (2- and 4-digit resolution) were successfully imputed with information score r^2^ > 0.3 and available for analysis. Due to the strong linkage disequilibrium between class II A1 and B1 loci, we present results only for each of the B1 loci (*HLA-DQB1*, *HLA-DRB1*, and *HLA-DPB1*).

Heterozygosity and homozygosity at each HLA locus and the number of heterozygous genotypes at class I loci (*A*, *B*, *C*) and class II loci (*DQB1*, *DRB1*, *DPB1*) were quantified using the imputed 4-digit resolution alleles. For each HLA locus, individuals were coded as homozygous (for any allele) or heterozygous, determined from the imputed alleles based on 4-digit resolution. To examine the joint effect of class I and class II loci, we combined the total number of heterozygotes for all loci and categorized into 3 groups: 0 to 1 heterozygote at all loci, 2 to 5 heterozygotes, or 6 heterozygotes. Subjects with 0 or 1 heterozygous genotype for all loci were used as reference group for subsequent analyses.

### HLA diversity and T cell features

To investigate the associations between germline HLA diversity and T cell features in tumors, we examined 2,839 patients of the 5,406 CRC cases who underwent pathology review for quantification of tumor infiltrating lymphocytes (TILs). Tumors were classified into two groups (TILs/high power field (hpf)>=2 or TILs/hpf <2) (18). Another subset of 2,357 patients from the 5,406 MECC cases with sufficient DNA macrodissected from bulk colorectal tumor tissues (2,335 formalin-fixed paraffin embedded and 22 frozen tissues) underwent survey-level T cell receptor (TCR) immunosequencing using immunoSEQ (Adaptive Biotechnologies, Seattle, WA; assay versions V2 and V4). ImmunoSEQ utilizes a multiplex PCR system to amplify hypervariable complementarity determining region 3β (CDR3β) sequences of the *TRB* gene (T cell receptor beta locus; https://www.genenames.org/data/gene-symbol-report/#!/hgnc_id/HGNC:12155). The number of unique rearrangements identified from our tissue samples ranged from 4 to 6,209. Detailed methods were reported elsewhere (19). In summary, TCR abundance (i.e. fraction_productive_of_cells) was estimated using the normalized number of productive *TRB* reads divided by the estimate of the total number of cells. We performed log transformation of TCR abundance due to the right-skewed distributions of the raw data. Productive simpson clonality (i.e. TCR clonality) was calculated as the square root of Simpson’s diversity index for all productive rearrangements for each sample. This metric scored TCR clonality from high to low, with high clonality indicating few unique clones and low clonality indicating a diverse T cell repertoire. Because two versions of the immunoSEQ assay, V2 (N=1,024) and V4 (N=1,333) were used, we calculated a z-transformation for TCR clonality for each sample based on the distribution of samples run on the same assay version.

### Statistical analysis

Unconditional logistic regression was used to estimate the association between HLA locus heterozygosity and CRC. Odds ratios (OR) and 95% confidence intervals (CIs) were calculated. Individuals with homozygous genotypes for each locus served as the reference category, and analyses were adjusted for sex, age, genotyping platform, and global ancestry (PC1-PC5). P values for trend tests were calculated by modeling the number of heterozygotes as an ordinal variable in the logistic regression, with individuals with homozygous genotypes at all loci as the reference group. To further evaluate the importance of microsatellite instability (MSI) in relation to HLA diversity, we stratified CRC cases by the MSI status of tumors (MSI-High (MSI-H) or microsatellite stable (MSS)) and compared with all controls to evaluate the associations between HLA diversity and CRC with or without this tumor molecular feature. Linear regression was used to evaluate the associations between HLA heterozygosity and each quantitative immunosequencing variable (TCR clonality and abundance). Logistic regression was conducted to examine the association between HLA diversity and pathology-based TILs. All regression models were adjusted for the factors listed above. All statistical analyses were performed using SAS 9.4 (SAS Institute). All tests of statistical significance were two-sided.

## Results

**Table 1** shows the distribution of demographic and clinical characteristics in the 5,406 CRC cases and 4,635 controls from the MECC study. On average, cases were 68 years old, which was similar to the mean age of 71 in the controls. Our study population comprised 52% males and 48% females, and around 58% are of Ashkenazi Jewish descent. A detailed description of the study population has been published elsewhere previously (13).

There were no significant associations between each of the HLA class I loci and CRC risk. Approximately 90% of the subjects were heterozygous for *HLA-A*, *HLA-B*, and *HLA-C*, respectively. However, in the joint analysis of all three HLA class I loci (*HLA-A*, *-B*, and -*C*) together, there was a 26% reduction in the odds of developing CRC for subjects with heterozygous genotypes at all 3 loci when compared to those with all homozygous genotypes (OR: 0.74, 95% Confidence Interval (CI): 0.56-0.97, *p*=0.0314; **Table 2**). However, no statistically significant linear trend was identified between the number of heterozygous HLA class I genotypes and CRC risk (*p*_trend_ = 0. 9168).

**Table 2:** Association between heterozygosity at HLA Class I loci (HLA-A, -B, and –C), Class II loci (HLA-DRB1, DQB1, and DPB1) and susceptibility to colorectal cancer.

Categories		Cases	%	Controls	%	Odds Ratio (95% CI)	*p* value
Class I Locus							
*HLA-A*	Heterozygote	4884	90.34	4171	89.99	1.02 (0.89-1.17)	0.7362
	Homozygote	522	9.66	464	10.01	1.00	
*HLA-B*	Heterozygote	4986	92.23	4293	92.62	0.94 (0.8-1.09)	0.4119
	Homozygote	420	7.77	342	7.38	1.00	
*HLA-C*	Heterozygote	4762	88.09	4066	87.72	1.04 (0.92-1.17)	0.5664
	Homozygote	644	11.91	569	12.28	1.00	
Total number of heterozygous Class I loci							
	3	4346	80.39	3684	79.48	0.74 (0.56-0.97)	0.0314^*^
	2	676	12.5	619	13.35	0.68 (0.51-0.91)	0.0101^*^
	1	242	4.48	240	5.18	0.64 (0.46-0.88)	0.0068^*^
	0	142	2.63	92	1.98	1.00	
						*p*_trend_ =	0.9168
Class II Locus							
*HLA-DRB1*	Heterozygote	4914	90.9	4214	90.92	0.97 (0.84-1.12)	0.6884
	Homozygote	492	9.1	421	9.08	1.00	
*HLA-DQB1*	Heterozygote	4600	85.09	3951	85.24	0.99 (0.88-1.11)	0.8371
	Homozygote	806	14.91	684	14.76	1.00	
*HLA-DPB1*	Heterozygote	3978	73.58	3481	75.1	0.92 (0.84-1.01)	0.0968
	Homozygote	1428	26.42	1154	24.9	1.00	
Total number of heterozygous Class II loci							
	3	3441	63.65	2974	64.16	0.75 (0.60-0.95)	0.0155^*^
	2	1403	25.95	1199	25.87	0.74 (0.58-0.94)	0.0145^*^
	1	363	6.71	326	7.03	0.73 (0.56-0.96)	0.0242^*^
	0	199	3.68	136	2.93	1.00	
						*p*_trend_ =	0.2328
Total number of heterozygous Class I or Class II loci							
	6	2880	53.27	2440	52.64	0.66 (0.49-0.87)	0.0038^**^
	2-5	2386	44.14	2112	45.57	0.61 (0.46-0.82)	0.0008^**^
	0-1	140	2.59	83	1.79	1.00	
						*p*_trend_ =	0.9077

^*^ p< 0.05, ^**^ p< 0.005.

Similarly, there were no significant associations between each of the HLA class II loci and CRC risk. Roughly 91%, 85% and 74% of the subjects were heterozygous for *HLA-DRB1*, *HLA-DQB1*, and *HLA-DPB1*, respectively. However, joint analyses suggested a 25% decreased odds of developing CRC for subjects with 3 heterozygotes at HLA-II loci as compared to those with all homozygotes at class II loci (OR: 0.75, 95% CI: 0.60-0.95, p=0.0155, *p*_trend_ = 0.2328; **Table 2**).

In joint analyses for class I and II loci, individuals with 2 to 5 heterozygous genotypes at HLA class I or II loci had significantly decreased odds of developing CRC (OR: 0.61, 95% CI: 0.46-0.82, *p*=0.0008) when compared to those with 0 or one heterozygous genotype. Moreover, individuals with all heterozygous genotypes at class I or II loci were at significantly lower odds of developing CRC (OR: 0.66, 95% CI: 0.49-0.87, *p*=0.0038, **Table 2**), when compared to those with no or one heterozygous genotype. We did not observe a significant linear dose-response relationship in the evaluation of the association between the number of heterozygous genotypes and CRC (*p*_trend_ = 0.9077).

### Stratified analyses by MSI

When restricting to 2,857 cases with microsatellite stable tumors, we observed a 34% decreased odds of developing CRC for individuals with heterozygous genotypes at all 3 HLA class I loci (OR: 0.66, 95% CI: 0.49-0.90, *p*=0.0094, **Table 3**). Subjects with 3 heterozygotes at HLA class II loci combined were at a reduced odds of CRC when compared to those with all homozygous HLA class II genotypes (OR: 0.67, 95% CI: 0.51-0.86, *p*=0.0022, *p*_trend_ = 0.0305). In the joint analysis for HLA class I and II loci, there was a 42% decreased odds of developing CRC among individuals with six heterozygous genotypes when compared to those with zero or one heterozygous genotypes (OR: 0.58, 95% CI: 0.42-0.80, *p*=0.0009, *p*_trend_ = 0.5767).

**Table 3:** Association of heterozygosity at HLA Class I and Class II loci and susceptibility to colorectal cancer stratified by microsatellite instability status of CRC cases.

Variable	MSS cases (N=2857) vs. All controls (N=4635)						MSI-H cases (N=621) vs. All controls (N=4635)					
	Cases	%	Controls	%	OR (95% CI)	*p* value	Cases	%	Controls	%	OR (95% CI)	*p* value
Class I loci												
*HLA-A*												
Heterozygote	2561	89.64	4171	89.99	0.95 (0.81-1.12)	0.5659	571	91.95	4171	89.99	1.22 (0.90-1.66)	0.2078
Homozygote	296	10.36	464	10.01	1.00		50	8.05	464	10.01	1.00	
HLA-B												
Heterozygote	2635	92.23	4293	92.62	0.94 (0.78-1.13)	0.5094	580	93.4	4293	92.62	1.14 (0.81-1.6)	0.463
Homozygote	222	7.77	342	7.38	1.00		41	6.6	342	7.38	1.00	
HLA-C												
Heterozygote	2515	88.03	4066	87.72	1.05 (0.91-1.22)	0.5114	551	88.73	4066	87.72	1.11 (0.85-1.45)	0.4589
Homozygote	342	11.97	569	12.28	1.00		70	11.27	569	12.28	1.00	
Total number of heterozygous class I loci												
3	2292	80.22	3684	79.48	0.66 (0.49-0.90)	0.0094^*^	519	83.57	3684	79.48	0.69 (0.4-1.17)	0.1634
2	355	12.43	619	13.35	0.60 (0.43-0.83)	0.0025^**^	61	9.82	619	13.35	0.48 (0.27-0.87)	0.0150^*^
1	125	4.38	240	5.18	0.55 (0.38-0.81)	0.0023^**^	23	3.7	240	5.18	0.47 (0.24-0.92)	0.0268^*^
0	85	2.98	92	1.98	1.00		18	2.9	92	1.98	1.00	
					*p*_trend_ =	0.8430					*p*_trend_ =	0.2208
Class II loci												
*HLA-DRB1*												
Heterozygote	2563	89.71	4214	90.92	0.84 (0.72-0.99)	0.0417^*^	575	92.59	4214	90.92	1.21 (0.88-1.67)	0.2381
Homozygote	294	10.29	421	9.08	1.00		46	7.41	421	9.08	1.00	
HLA-DQB1												
Heterozygote	2405	84.18	3951	85.24	0.92 (0.80-1.05)	0.1973	530	85.35	3951	85.24	1.01 (0.8-1.29)	0.9088
Homozygote	452	15.82	684	14.76	1.00		91	14.65	684	14.76	1.00	
HLA-DPB1												
Heterozygote	2091	73.19	3481	75.1	0.92 (0.83-1.03)	0.1623	449	72.3	3481	75.1	0.89 (0.74-1.08)	0.2485
Homozygote	766	26.81	1154	24.9	1.00		172	27.7	1154	24.9	1.00	
Total number of heterozygous class II loci												
3	1798	62.93	2974	64.16	0.67 (0.51-0.86)	0.0022^**^	391	62.96	2974	64.16	0.79 (0.49-1.27)	0.3304
2	725	25.38	1199	25.87	0.64 (0.49-0.84)	0.0012^**^	173	27.86	1199	25.87	0.82 (0.5-1.33)	0.4222
1	215	7.53	326	7.03	0.73 (0.54-1.00)	0.0490^*^	35	5.64	326	7.03	0.65 (0.37-1.17)	0.1518
0	119	4.17	136	2.93	1.00		22	3.54	136	2.93	1.00	
					*p*_trend_ =	0.0305^*^					*p*_trend_ =	0.8597
Total number of heterozygous Class I or Class II loci												
6	1496	52.36	2440	52.64	0.58 (0.42-0.80)	0.0009^**^	342	55.07	2440	52.64	0.63 (0.37-1.07)	0.0893
2-5	1278	44.73	2112	45.57	0.55 (0.40-0.76)	0.0003^**^	261	42.03	2112	45.57	0.53 (0.31-0.92)	0.0231^*^
0-1	83	2.91	83	1.79	1.00		18	2.9	83	1.79	1.00	
					*p*_trend_ =	0.5767					*p*_trend_ =	0.3751

^*^ p< 0.05, ^**^ p< 0.005.

MSS, microsatellite stable; MSI-H, microsatellite instable.

Analyses on a smaller subset of 621 cases with MSI tumors were conducted, and similar strengths and directions of associations between zygosity of HLA class I and II loci and CRC risk were observed as in the overall analysis with all cases. However, the results did not reach statistical significance, potentially due to the smaller sample sizes (**Table 3**).

### HLA heterozygosity and T cell features

To further examine if germline HLA diversity is associated with tumor T cell landscapes, we conducted analyses limited to a subset of 2,357 CRC patients who underwent immunosequencing and 2,839 CRC patients with pathology-based TIL scoring. We found that patients with more heterozygote HLA class I and/or II genotypes generally displayed lower clonality (higher diversity) and higher abundance in their tumor T cell repertoires (**Supplementary Tables 1** and **2**). Similarly, patients with more heterozygotes in class I and II alleles were likely to manifest higher TILs in their tumors (**Supplementary Table 3**). Having a heterozygous genotype at *DRB1* or *DQB1* was significantly associated with higher TILs (OR: 1.74 and 1.44, *p*=0.003 and 0.0024, respectively). The presence of either 2 or 3 heterozygous genotypes for class II alleles was marginally associated with higher TILs (*p*=0.0650 and 0.0789, respectively; *p*_trend_ = 0.0161). Results also suggested that CRC patients with all 6 heterozygous genotypes at class I and II were likely to have higher TILs (*p*=0.1006). However, these results did not reach statistical significance.

## Discussion

Using the largest dataset of imputed HLA genotypes in CRC to date, we demonstrated that germline genetic diversity at HLA class I and II loci is associated with reduced CRC risk. Individuals with more diverse genotypes (more heterozygotes) in HLA class I and/or II loci bear a reduced risk of developing CRC when compared to those with less diverse genotypes (homozygotes at all HLA class I and/or II loci). This increase in HLA diversity remained statistically significantly associated with reduced risk of CRC even when we restricted to MSS CRC cases.

It has been suggested that pathogen-mediated selection is the driving force maintaining diversity at the HLA loci. Heterozygote advantage, one of the proposed mechanisms of pathogen-mediated selection, was originally described in infectious and autoimmune diseases, such as individuals with AIDS (3, 20), HBV and HCV infections (21), inflammatory bowel disease (7), and psoriatic arthritis (22). Individuals who are heterozygous at HLA loci are able to respond to a broader range of pathogen peptides than those who are homozygous, therefore sustaining efficient immune responses against a larger variety of pathogens (1).

A diverse germline HLA genotype can also affect tumor surveillance and shape the cancer genome. Marty at al. showed that individuals’ HLA class I and class II genotypes together impact the oncogenic mutational landscape in a complementary manner (23, 24). Using The Cancer Genome Atlas (TCGA), they developed a predictive tool based on HLA class I genotypes to evaluate the presentation of recurrent cancer mutations and demonstrated HLA class I-mediated immunoediting during tumor development influences the landscape of oncogenic mutations at a population level (23). Later, they illustrated that HLA class II genotypes, under the surveillance of CD4^+^ T cells, are also important for immunoediting in early cancer development (24). Having a stronger selective pressure on driver mutations in tumors, HLA class II plays an important role in early cancer development. Together, these results demonstrated that HLA class I and II genotypes are both involved in the immunoediting process in carcinogenesis by establishing the patterns of immune escape from both CD8^+^ and CD4^+^ T-cell responses in a complementary way. Similarly, rather than identifying specific HLA allelic associations in CRC, we showed that the combined total number of heterozygous genotypes at HLA class I and/or II is associated with decreased CRC risk, suggesting a potential complementary effect of HLA class I and class II diversity. Of note, because we did not observe a dose-response relationship between the number of heterozygous genotypes in HLA loci and CRC, it is likely that the association between HLA diversity and CRC risk follows a non-linear trend.

Heterozygote advantage for HLA class I and/or II in relation to cancer was first demonstrated in non-Hodgkin’s lymphoma (NHL), with distinct associations between class I and/or II homozygosity in different subtypes of non-Hodgkin’s lymphoma (8). While risk of diffuse large B-cell lymphoma and marginal zone lymphoma were increased with homozygosity of class I HLA-B and HLA-C loci and the class-II HLA-DRB1 locus, follicular lymphoma risk was associated with the increase in homozygous loci for HLA class II genes. The authors suggested a potential role for HLA zygosity in NHL etiology, and distinct immune pathways for the different NHL subtypes. Unlike NHL or other virus-associated diseases, we did not observe zygosity of any specific HLA alleles to be associated with risk of developing CRC. It is possible that the specific HLA allelic association simply was not observed in this population, or that non-exclusive HLA class-I and -II loci play an important role in CRC etiology. We did not observe individual 4-digit alleles to be statistically significantly associated with odds of developing CRC in our study population after multiple testing correction, potentially attributable to low power (data not shown). However, we demonstrated that HLA diversity in HLA class I and II loci combined is associated with reduced CRC risk. Our results, in line with the previous findings from Marty et al., implied that complementary functions of heterozygous HLA class I and/or II alleles in the antigen presenting machinery are more relevant for CRC than individual 4-digit alleles in either class.

With the growing body of literature, more research focused on germline HLA zygosity in cancer etiology has emerged. A recent study examined 17,405 cancer patients diagnosed with non-virus related solid tumors and 11,448 controls and found no evidence of an association between number of homozygotes at HLA class I, class II or class I and II loci and overall cancer risk (25). Colon cancer was one of the twelve non-virus associated tumors evaluated. However, the study included only 82 colon cancer patients and reported no significant association specific to this cancer type. Another pan-cancer analysis on HLA diversity and risk of 25 cancers using UK biobank has shown that the diversity of HLA class II is associated with a lower risk of lung cancer, head and neck cancer, and non-Hodgkin lymphoma (26). They also identified protective effects of HLA diversity in pathological subtypes with higher mutation burden, such as lung squamous cell carcinoma and diffuse large B cell lymphoma. However, there was no significant association observed for class I or class II diversity and risk for colon and rectal cancer among 3,232 colon and 2,035 rectal cancer patients. Our study is the first and largest population-based study with HLA imputed genotypes to investigate the role of HLA diversity in risk for developing CRC. With the large sample size, we were able to examine HLA diversity and CRC risk stratified by the MSI status of tumors, given that tumor escape mechanisms differ by MSI status. Individuals who have 6 heterozygous genotypes at HLA class I and II had a reduced odds of developing CRC regardless of MSI status. Slightly stronger associations between HLA classes I and/or II zygosity and CRC risk were observed in participants with MSS tumors than the overall case set, indicating that our initial observations were not driven by MSI-H tumors. Findings similar to the MSS group were observed in MSI group. However, the associations did not reach statistical significance; this may be due to small sample size (MSI-H tumors are 11.27% of the full case dataset). Additional studies with larger numbers of patients with MSI tumors are needed to replicate the results.

In our analysis of a subset of CRC patients on HLA heterozygosity and tumor T cell features, we found that having more heterozygotes in germline HLA class I and/or class II alleles may be associated with a more diverse T cell receptor repertoire, higher TCR abundance and tumor infiltrating lymphocytes in tumors. Although the results were not statistically significant, possibly due to smaller sample sizes or a relatively weak effect, the directions of associations are in line with our hypothesis where germline HLA diversity affects tumor immune surveillance, and consequently, reduces CRC risk (**Figure 1**). Nonetheless, studies with larger sample sizes are warranted to provide more support on the relationship between HLA diversity and T cell landscapes in CRC.

Having a diverse HLA system may also be relevant to response to immune checkpoint inhibitor (ICI) treatments. Several recent studies have focused on HLA class I diversity and the response to immune checkpoint inhibitors. HLA class-I diversity measured by HLA evolutionary divergence (HED) has been associated with better response to immunotherapy in advanced-stage melanoma or non-small cell lung cancer (11). Other studies in gastrointestinal (including CRC), non-small cell lung and kidney cancer have observed similar results where cancer patients with higher HLA class-I divergence are more responsive to ICI treatments (27–29). However, a recent meta-analysis with more than 1,000 patients underwent ICI across seven tumor types failed to report HLA evolutionary divergence as a predictor for ICI treatment (30). Very few CRC patients in our study received ICI because they were recruited by 2017; therefore, we were unable to examine the association between HLA diversity and response to immunotherapy. More research is needed to understand the implications of HLA diversity to ICI response in CRC. Further, expanded datasets are needed to powerfully examine the treatment-independent prognostic relevance of HLA diversity.

Our study is the largest population-based study to examine HLA diversity and CRC risk to date. It revealed for the first time that heterozygosity at HLA class I and/or II loci confers decreased risk for CRC. These findings highlight the potential role of germline HLA diversity in CRC susceptibility. Our study had a few key limitations. First, misclassification of HLA alleles from imputation is possible. However, other studies using four-digit HLA genotype data imputed from SNP2HLA have shown that the concordance rate between imputed and directly genotyped HLA data is greater than 95% in Caucasian populations using T1DGC as a reference population (17). Second, because our study subjects were mostly of European ancestry, these results cannot necessarily be generalized to other ancestral groups. Studies from other racial or ethnic groups are warranted to investigate the role of HLA diversity in the biologic mechanisms for CRC in different populations. Third, TCR clonality metrics can be skewed or more difficult to interpret when there is a lower number of rearrangements; however, productive Simpson clonality is nonetheless a valuable starting to point to begin examining the relationship between HLA diversity and tumor-associated T cell responses. Finally, larger datasets will be needed to analyze the zygosity of specific HLA alleles associated with CRC risk.

In summary, our findings support a heterozygote advantage for HLA class I and II loci in reducing CRC susceptibility. This underscores an important role for germline HLA genetic variability in the etiology of CRC, potentially operating through a mechanism of increased diversity of tumor neoantigens that can be displayed to the adaptive immune system.

## Data availability statement

The datasets presented in this study can be found in online repositories. The names of the repository/repositories and accession number(s) can be found below: https://www.ncbi.nlm.nih.gov/gap/, phs001045.v1.p1 https://www.ncbi.nlm.nih.gov/gap/, phs001856.v1.p1 https://www.ncbi.nlm.nih.gov/gap/, phs001903.v1.p1.

## Ethics statement

The MECC study protocol was approved by the City of Hope Institutional Review Board (Protocol Number 19404). The studies were conducted in accordance with the local legislation and institutional requirements. The participants provided their written informed consent to participate in this study.

## Author contributions

Y-YT: Conceptualization, Formal Analysis, Investigation, Methodology, Visualization, Writing – original draft, Writing – review & editing. CQ: Conceptualization, Investigation, Writing – review & editing. JB: Investigation, Project administration, Writing – review & editing. RS: Visualization, Writing – review & editing. SL: Investigation, Project administration, Writing – review & editing. MM: Investigation, Writing – review & editing. KM: Project administration, Writing – review & editing. GI: Project administration, Writing – review & editing. CW: Investigation, Project administration, Writing – review & editing. KT: Investigation, Writing – review & editing. DD: Conceptualization, Writing – review & editing. FM: Writing – review & editing. AM: Writing – review & editing. HR: Investigation, Project administration, Writing – review & editing. WK: Conceptualization, Writing – review & editing. JG: Investigation, Project administration, Writing – review & editing. VM: Funding acquisition, Methodology, Supervision, Writing – review & editing. GR: Investigation, Project administration, Supervision, Writing – review & editing. SG: Conceptualization, Funding acquisition, Investigation, Project administration, Supervision, Writing – review & editing. SS: Conceptualization, Funding acquisition, Investigation, Methodology, Supervision, Writing – original draft, Writing – review & editing.
