# Supplementary material for: Heterozygote advantage at HLA class I and II loci and reduced risk of colorectal cancer
Source: Front Immunol. 2023 Oct 24;14:1268117. doi: 10.3389/fimmu.2023.1268117 (PMC10627840; doi:10.3389/fimmu.2023.1268117)
Supplement: Supplementary file 1 [file Table_1.docx]

***Supplementary Material***

**Title: Heterozygote advantage at HLA class I and II loci and reduced risk of colorectal cancer**

Ya-Yu Tsai, Chenxu Qu, Joseph D. Bonner, Rebeca Sanz-Pamplona, Sidney S. Lindsey, Marilena Melas, Kevin J. McDonnell, Gregory E. Idos, Christopher P. Walker, Kevin K. Tsang, Diane Da Silva, Ferran Moratalla, Asaf Maoz, Hedy S. Rennert, W. Martin Kast, Joel K. Greenson, Victor Moreno, Gad Rennert, Stephen B. Gruber, Stephanie L. Schmit^*^

* **Correspondence:** Stephanie L. Schmit: Email: [schmits3@ccf.org](mailto:schmits3@ccf.org)

Supplementary Table 1. Association between T cell receptor clonality and HLA heterozygosity in 2,357 MECC cases. Linear regression models were adjusted for sex, age at diagnosis, genotyping platform/batch, and PC1 to PC5.

| **Category** | **Estimate** | **Standard Error** | **Odds Ratio (95% Confidence Interval)** | ***p* Value** |
| --- | --- | --- | --- | --- |
| Total number of heterozygous Class I loci | | | | |
| 3 | -0.13 | 0.13 | 0.88 (0.68-1.12) | 0.2936 |
| 2 | -0.22 | 0.14 | 0.8 (0.61-1.05) | 0.1132 |
| 1 | 0.03 | 0.16 | 1.03 (0.76-1.41) | 0.8384 |
| 0 |  |  | 1.00 |  |
| Total number of heterozygous Class II loci | | | | |
| 3 | -0.02 | 0.11 | 0.98 (0.79-1.21) | 0.8415 |
| 2 | 0.10 | 0.11 | 1.1 (0.89-1.37) | 0.3828 |
| 1 | 0.04 | 0.13 | 1.04 (0.81-1.34) | 0.7506 |
| 0 |  |  | 1.00 |  |
| Total number of heterozygous Class I or Class II loci | | | | |
| 6 | -0.09 | 0.13 | 0.92 (0.71-1.18) | 0.5032 |
| 2-5 | -0.06 | 0.14 | 0.95 (0.72-1.24) | 0.6796 |
| 0-1 |  |  | 1.00 |  |

Supplementary Table 2. Association between T cell receptor abundance and HLA heterozygosity in 2,357 MECC cases. Linear regression models were adjusted for sex, age at diagnosis, genotyping center/platform, and PC1 to PC5.

| **Category** | **Estimate** | **Standard Error** | **Odds Ratio (95% Confidence Interval)** | ***p* Value** |
| --- | --- | --- | --- | --- |
| Total number of heterozygous Class I loci | | | | |
| 3 | 0.18 | 0.14 | 1.20 (0.91-1.58) | 0.2052 |
| 2 | 0.28 | 0.15 | 1.32 (0.98-1.79) | 0.0687 |
| 1 | 0.02 | 0.18 | 1.02 (0.72-1.44) | 0.9103 |
| 0 |  |  | 1.00 |  |
| Total number of heterozygous Class II loci | | | | |
| 3 | 0.20 | 0.12 | 1.22 (0.97-1.54) | 0.0923 |
| 2 | 0.14 | 0.12 | 1.15 (0.90-1.46) | 0.2673 |
| 1 | 0.18 | 0.14 | 1.20 (0.91-1.58) | 0.2073 |
| 0 |  |  | 1.00 |  |
| Total number of heterozygous Class I or Class II loci | | | | |
| 6 | 0.23 | 0.14 | 1.25 (0.95-1.66) | 0.1131 |
| 2-5 | 0.21 | 0.15 | 1.24 (0.92-1.67) | 0.1598 |
| 0-1 |  |  | 1.00 |  |

Supplementary Table 3. Association between pathology-based tumor infiltrating lymphocytes per high power field and HLA heterozygosity in 2,839 MECC cases. Linear regression models were adjusted for sex, age at diagnosis, genotyping center/platform, and PC1 to PC5.

| **Categories** | **Cases** | **%** | **Controls** | **%** | **Odds Ratio (95% CI)** | ***p* value** |
| --- | --- | --- | --- | --- | --- | --- |
| **Class I Locus** |  |  |  |  |  |  |
| *HLA-A* |  |  |  |  |  |  |
| Heterozygote | 781 | 91.13 | 1785 | 90.06 | 1.12 (0.85-1.48) | 0.4143 |
| Homozygote | 76 | 8.87 | 197 | 9.94 | 1.00 |  |
| *HLA-A* |  |  |  |  |  |  |
| Heterozygote | 792 | 92.42 | 1839 | 92.79 | 0.95 (0.7-1.29) | 0.7325 |
| Homozygote | 65 | 7.58 | 143 | 7.21 | 1.00 |  |
| *HLA-C* |  |  |  |  |  |  |
| Heterozygote | 757 | 88.33 | 1756 | 88.6 | 0.99 (0.77-1.27) | 0.9212 |
| Homozygote | 100 | 11.67 | 226 | 11.4 | 1.00 |  |
| Total number of heterozygous Class I loci | | | | | | |
| 3 | 696 | 81.21 | 1615 | 81.48 | 1.19 (0.71-1.98) | 0.5086 |
| 2 | 102 | 11.9 | 225 | 11.35 | 1.24 (0.71-2.17) | 0.4432 |
| 1 | 38 | 4.43 | 85 | 4.29 | 1.25 (0.66-2.36) | 0.4899 |
| 0 | 21 | 2.45 | 57 | 2.88 | 1.00 |  |
|  |  |  |  |  | *P* _trend_= | 0.8591 |
| **Class II Locus** |  |  |  |  |  |  |
| *HLA-DRB1* |  |  |  |  |  |  |
| Heterozygote | 797 | 93 | 1754 | 88.5 | 1.74 (1.29-2.35) | 0.0003^**^ |
| Homozygote | 60 | 7 | 228 | 11.5 | 1.00 |  |
| *HLA-DQB1* |  |  |  |  |  |  |
| Heterozygote | 748 | 87.28 | 1638 | 82.64 | 1.44 (1.14-1.81) | 0.0024^**^ |
| Homozygote | 109 | 12.72 | 344 | 17.36 | 1.00 |  |
| *HLA-DPB1* |  |  |  |  |  |  |
| Heterozygote | 619 | 72.23 | 1458 | 73.56 | 0.94 (0.79-1.13) | 0.5127 |
| Homozygote | 238 | 27.77 | 524 | 26.44 | 1.00 |  |
| Total number of heterozygous Class II loci | | | | | | |
| 3 | 554 | 64.64 | 1226 | 61.86 | 1.51 (0.97-2.34) | 0.0650 |
| 2 | 227 | 26.49 | 506 | 25.53 | 1.50 (0.95-2.37) | 0.0789 |
| 1 | 48 | 5.6 | 160 | 8.07 | 1.02 (0.6-1.75) | 0.9313 |
| 0 | 28 | 3.27 | 90 | 4.54 | 1.00 |  |
|  |  |  |  |  | *P* _trend_= | 0.0161^*^ |
| Total number of heterozygous Class I or Class II loci | | | | | | |
| 6 | 464 | 54.14 | 1043 | 52.62 | 1.57 (0.92-2.7) | 0.1006 |
| 2-5 | 375 | 43.76 | 878 | 44.3 | 1.51 (0.88-2.6) | 0.1342 |
| 0-1 | 18 | 2.1 | 61 | 3.08 | 1.00 |  |
|  |  |  |  |  | *P* _trend_= | 0.2520 |

^*^ p< 0.05

^**^ p< 0.005
